# Early Reduction of the Posterior Column: A Surgical Technique in AO/OTA C3 Tibial Pilon Fractures

**DOI:** 10.3390/jpm13030551

**Published:** 2023-03-20

**Authors:** Yanchun Gao, Hongyi Zhu, Yanjie Guo, Xingang Yu

**Affiliations:** Department of Orthopedic Surgery, Shanghai Jiao Tong University Affiliated Sixth People’s Hospital, Shanghai Jiao Tong University, Shanghai 200233, China

**Keywords:** trauma, staged treatment, tibial pilon fractures

## Abstract

Staged treatment for pilon fractures is widely accepted. It remains to be discussed how to reduce and fix posterior column fractures while avoiding clinical complications. We provided a staged treatment protocol with detailed surgical techniques for closed AO Foundation/Orthopaedic Trauma Association (AO/OTA) C3 tibial pilon fractures with fibular fractures. In the first stage, the internal fixation of the fibula and distal tibial posterior column is accompanied by an external fixator. After swelling, the medial and anterior columns were fixed via the posteromedial approach in the second stage. We advocate early reduction and fixation of the posterior column and lateral column. The right timing of surgery can ensure well-reduced articular surface and alignment while minimizing soft tissue complications.

## 1. Background

Pilon fractures, accounting for 4–10% of all tibial fractures, are a severe challenge to surgeons due to poor prognosis [1,2]. According to AO Foundation/Orthopaedic Trauma Association (AO/OTA) classification, a C3-type pilon fracture is defined as a comminuted fracture of the articular surface of the distal tibia involving the metaphysis and diaphysis. It is often accompanied by severe soft tissue injury [3]. Fibula fractures accompany about 75–90% of C3 pilon fractures. Due to axial violence, comminuted fractures often occur on the articular surface and metaphysis of the distal tibia, often accompanied by damage to the surrounding soft tissue. 

The purpose of surgical treatment is to restore ankle function by fracture fixation, which acquires a high-quality reduction of articular surfaces and restoration of tibial alignment. The reduction of comminuted articular fractures places high demands on the surgeon’s surgical skills. In addition, complex pilon fractures suggest high-energy injury, accompanied by increased complications such as infection, marginal necrosis, or poor wound healing [4,5].

Staged treatment for AO/OTA C3 pilon fractures has long been widely accepted [6,7,8,9,10]. However, the details of the surgery remain controversial, including whether to use early partial plate fixation, the choice of surgical time, choice of surgical approach, and repositioning techniques. Some surgeons advocate early fixation of the fibula for pilon fractures with fibula fractures. In contrast, others believe that the open internal fixation should be performed after the complete soft tissue detumescence [11]. Longer immobilization times provide good soft tissue detumescence, but bone resorption and osteoporosis increase the difficulty of surgical reduction [10,12]. The single anterior incision has effectively exposed and fixed the distal tibia fragment. Still, the inability to expose and reduce the posterior malleolus fragment has always been an unavoidable disadvantage [13,14]. The combined anterior and posterior approaches can effectively reduce and fix the posterior column. However, it was pointed out that excessive soft tissue dissection and double incision increased the risk of the flap and soft tissue [15,16].

This study used a staged treatment of external fixation combined with partial internal fixation to treat AO/OTA C3 pilon fractures with fibular fractures. We have gained new insights into the early fixation of the posterior column and shared surgical techniques that will contribute to clinical outcomes.

## 2. Methods

### 2.1. Preoperative Preparation

The tibiofibular X-ray, including knee and ankle joints and CT scan of the ankle joint with three-dimensional reconstruction, were completed in the emergency room to evaluate fracture line distribution, fracture displacement, and comminution. Calcaneal traction was given under local anesthesia. Ice compress and dehydration treatment were shown to reduce edema. The preoperative preparation time of the first surgery ranged from 2 to 5 days after injury (mean 2.7 days). 

### 2.2. Surgical Technique

#### 2.2.1. Stage I

In the stage I surgery, the patient underwent a posterolateral approach between the distal fibula and the Achilles tendon in a lateral position. The superficial peroneal and sural nerves were protected, and the fibula fracture was exposed. Soft tissues and blood clots are removed from the fibular fracture site. With the help of repositioning forceps, the fibula was anatomically reduced and fixed with an anatomical locking plate lateral to the fibula. Restoring fibula length and alignment is critical during the surgery for comminuted fibula fractures [17,18,19].

**Figure 1:**
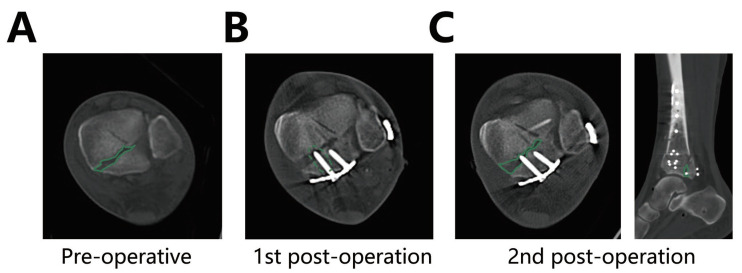
Inappropriate length of the screws impedes Volkmann fragment repositioning. (**A**) The CT scan shows the morphology of the preoperative pilon fracture bone fragments. (**B**) The posterior screws should be shorter than the size of the posterior ankle fragment. (**C**) CT scan of the ankle after the second surgery showed that an excessively long posterior screw impeded the reduction of the anterolateral bone fragments during the second surgery.

Exposure of the distal tibia and metaphyseal segments is very important. The conjunctions of the metaphyseal cortex determined the anatomic reduction of the posterior column. Kirschner wires temporarily fixed the posterior fragment. Subsequently, a T-type or straight locking plate was placed close to the posterior column, and short screws were used in the distal of the plates to fix the posterior fragments. Plates provided an effective buttress, and short screws were used to improve the posterior malleolus. When excessively long posterior screws break through the anterior edge of the posterior fragment, the screws will prevent the reduction during secondary surgery. Therefore, preoperative CT transverse images are measured to estimate the appropriate length of the screws in the Volkmann fragment (Figure 1). The wound was closed, and negative pressure drainage was left postoperatively. Then, the lateral calcaneus and proximal medial tibia were drilled, two 5 mm Shanz pins were inserted, respectively, and an external fixator was installed across the ankle joint. The patients were routinely treated with anti-inflammatory and detumescent therapy after the operation.

#### 2.2.2. Stage II

After the soft tissue swelling had subsided entirely and skin wrinkles showed, the second phase of the operation was performed. 

**Figure 2:**
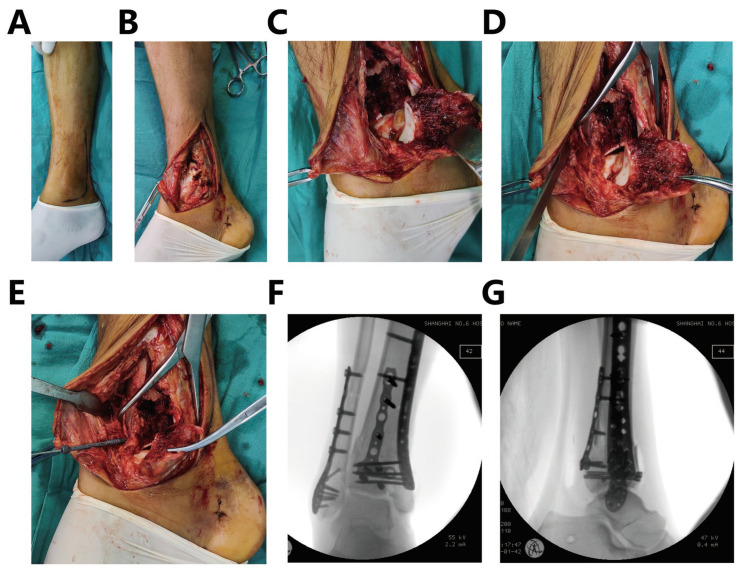
Intraoperative fragments and joint surface reduction procedure and intraoperative fluoroscopy. (**A**) A medial incision. (**B**) Full-thickness skin flap was lifted over the bone (**C**) The medial fragment was lifted distally along the original fracture line, and the medial and anterior columns were exposed. (**D**) The articular surface was reduced under direct vision and temporarily fixed with point-type reduction forceps. (**E**) Screws were implanted from front to back above the articular surface under direct vision. (**F**) Intraoperative fluoroscopy of anteroposterior view. (**G**) Intraoperative fluoroscopy of lateral view.

The external fixator was removed in a supine position three days before the second surgery. In stage II surgery, the procedure is performed using a medial incision in which the posterior edge of the tibia is past the medial ankle and extends forward (Figure 2A). The tarsal tunnel is not incised unless necessary and is protected throughout the procedure. The full-thickness skin flap was lifted over the bone, and the great saphenous vein was protected (Figure 2B). The medial fragment was lifted distally along the original fracture line, and then the medial and anterior columns were shown clearly (Figure 2C). The posterior column was reduced and fixed at the first surgery, while the mid-articular surface and anterior column were exposed under direct vision. The posterior column was used as a reference to reduce the collapsed middle articular surface and anterior column fragments. The articular surface was reduced under direct vision and temporarily fixed with point-type reduction forceps. The superior edge of the talus can also be used as a reference line for repositioning (Figure 2D). The large allogeneic bone or iliac bone was filled in the metaphyseal defect site. One to two screws were implanted from front to back above the articular surface under direct vision (Figure 2E). Subsequently, the medial column fragment was reduced and fixed with Kirschner wire, fixed by a medial anatomic plate of the distal tibia after the fracture reduction. Intraoperative fluoroscopy showed a well-reduced fracture with a flat joint surface (Figure 2F,G). The wound was flushed and closed, and drainage was left in place.

### 2.3. Postoperative Management

Patients were encouraged to initiate toe and ankle flexion and dorsiflexion exercises on the second day after surgery without external fixators or braces. Negative pressure suction was removed two days postoperatively. Weight-bearing walking was prohibited until radiology confirmed initial fracture healing. Partial weight-bearing walking was started gradually, 8–12 weeks postoperatively.

## 3. Results

From October 2016 to September 2019, 29 patients with closed complex pilon fractures combined with fibular fractures were treated in our hospital, with an average follow-up time of 25.9 months (ranging from 12 to 66 months). The average age was 54.1 years. According to Burwell–Charnley X-ray standards, the anatomical reduction was found in 21 patients, a good reduction in six patients, and a fair reduction in two patients. Post-operative imaging follow-up showed that most of the fractures healed within 2–3 months after surgery, with an average healing time of 3.38 months (ranging from 2 to 6 months). The postoperative function was assessed according to the AOFAS Ankle-Hindfoot scale. It showed that the average score was 82.4 points (range 62–94 points).

**Figure 3:**
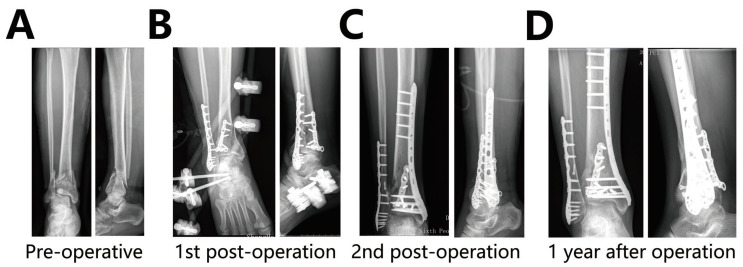
(**A**) Pre-operative X-ray. (**B**) The x-rays show the fixation of the posterior tibial column and fibula by a locking plate in the first surgery, combined with external fixator. (**C**) The x-ray shows the fixation of the tibia with a medial locking plate during the second surgery. (**D**) The x-ray shows the healing of the patient’s fracture one year after surgery.

Figure 3 demonstrates a 64 year old male with an AO/OTAC3 pilon fracture who achieved good fracture reduction following our staged treatment protocol. We performed his first surgery two days after the fracture. He underwent a second surgery 19 days later. According to Burwell–Charnley’s fracture reduction quality assessment, an anatomic reduction was achieved. The AOFAS score for the assessment of ankle function was 94, with a grade of Excellent.

**Figure 4:**
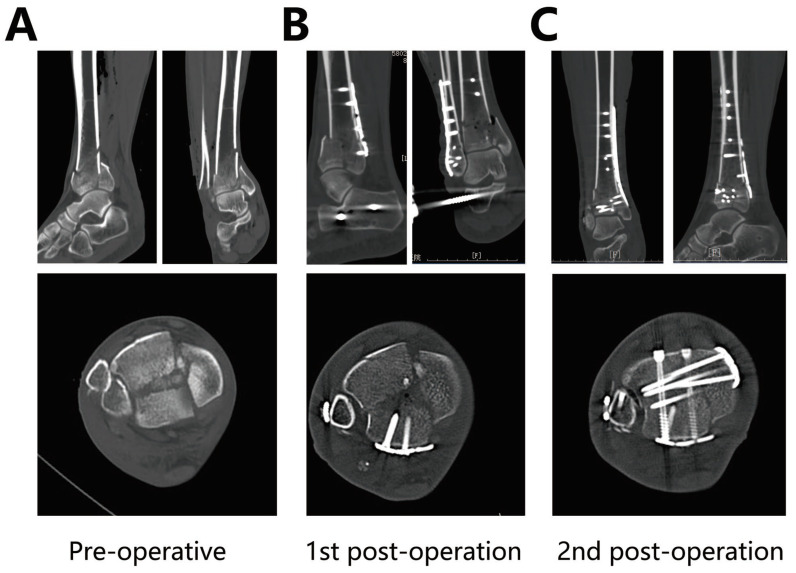
CT images of the patient (**A**) after the fracture (**B**) after the first surgery (**C**) after the second surgery.

Figure 4 shows a CT scan of a 58 year old male before and after the first and second surgery. We can see the process of fragment reduction during the process of this staged treatment.

## 4. Discussions

For AO/OTA C3 tibial pilon fractures with fibula fractures, the strategy of staged treatment has been widely accepted by most surgeons, significantly reducing the risk of soft tissue complications [20,21]. Nonetheless, the long preoperative waiting time may lead to bone loss, scarring, soft tissue contractures, increased difficulty in reducing fractures, and other complications. For the staged treatment of pilon fractures, selecting the time of the second surgery is a difficult part of the treatment decision. An excellent treatment should achieve a good reduction while avoiding soft-tissue complications, which improves fracture healing and ankle joint function.

Many researchers recommend using a temporary trans-ankle external fixator for approximately 1 to 4 weeks, followed by an open reduction and internal fixation after the surrounding soft tissues are repaired [22,23]. Despite careful soft-tissue management, the risk of wound complications was still relatively high compared with other orthopedic trauma surgeries [11,24]. Swelling reacts with a cascade of biological and biomechanical changes affecting the local circulation, inflammation, and immune reaction [25]. Ischemic necrosis, caused by the high tension of the flap or compression of displaced fracture fragments, leads to prolonged treatment or poor healing. Premature timing of the surgery often leads to difficult wound closure, poor wound healing, and muscle necrosis. In addition, scar hyperplasia, bone resorption, and callus formation can cause the loss of anatomical signs, increasing difficulties in the second surgery. Soft tissue contracture also makes it challenging for fracture reduction. These conditions suggest an early requirement for fracture reduction.

In addition to soft tissue issues, achieving anatomic reduction and fixation of the articular surface is still the key to treatment. Some studies have shown that the quality of fracture reduction may be significantly correlated with long-term functional prognosis [26,27]. The present study determined that fibular fixation was pivotal in treating pilon fractures, particularly those with severe metaphyseal comminution and joint involvement. Fibular fixation facilitated tibial reduction and helped maintain the appropriate length and alignment of the tibia [28]. Early reduction and fixation of the fibula facilitate the restoration of limb length, which provides lateral column support to the ankle fracture and contributes to the second-stage reduction of the tibial fracture. In addition, Ketz et al. proposed fibular reduction. Reduction and fixation of the fibula could restore alignment and rotation of the lateral column, simultaneously reducing the posterolateral (Volkmann) and anterolateral (Chaput) by extending the anterior and posterior tibiofibular ligaments [19]. The mal-reduction of the fibula will affect the reduction of the distal tibial fragments in secondary surgery. Therefore, the operator should pursue the anatomic reduction of the fibula in the first surgery to restore the length and force line of the ankle.

We want to clarify that, throughout the procedure (both the first and second stages), the articular surface is repositioned based on the principle of “back to the forward.” In the first stage, the posterior column is reduced to provide the anatomical landmarks for the second stage. Based on this, the articular surfaces of the middle and anterior columns are repositioned one by one. If the fragments of the articular surface are not reduced in the first stage, the second-stage reduction becomes very difficult [13]. Ketz (2012) and Sanders (2021) reported that the loss of joint reduction was less than 2 mm in one-stage direct reduction posterior column fractures. These studies demonstrate that early removal of the posterior tibial column is reliable for restoring articular surface flattening [19,29]. For posterior column fractures with metaphyseal comminution, a pre-contorted T-shaped or straight locking plate can effectively fix the fracture, especially for shear fractures of the posterior malleolus. For posterior column fractures, the reduction of the metaphysis should precede the reduction of the posterior malleolus, which turns a C-type fracture into a B-type fracture, while reducing the limb alignment also provides a good reference mark for reducing the anterior and medial columns in the second-stage operation. We recommend an intraoperative assessment to prevent poor posterior column reduction and to apply sufficient pressure on the articular surface to ensure absolute stability of the fracture fixation.

In the treatment of complex pilon fractures, different surgical approaches have their advantages [30,31,32]. For AO/OTA C3 pilon fractures, single-incision surgery is insufficient for the reduction and fixation of the posterior column, and double incision shows its irreplaceable superiority in the reduction of the articular surface [8,14,16,33,34]. For dual incisions, insufficient flap width is an essential factor leading to skin necrosis, especially in patients with preoperative soft tissue contusion [35]. Even after adequate swelling and the appearance of skin wrinkling, the combined anterior–posterior approach in the treatment of pilon fractures inevitably faces the risk of difficulty in wound closure, flap necrosis, or infection [35,36]. Delayed wound closure may increase the risk of wound infection and decrease joint function [37]. In this study, staged treatment provides sufficient time to restore blood supply to the dissected tissue and skin after fixation of the posterior column by a posterolateral approach.

Many surgeons use an anterior approach to expose the anterior and part of the mid-articular surface of the tibiotalar joint [38]. However, when the anterior incision is used to reduce the fracture of the medial and anterior column, the exposure and fixation of the medial column are limited [14,39]. In our second-stage surgery, the posterior column was used as a reference to reduce the collapsed articular surface. The mid-articular surface and anterior column were exposed and fixed by a medial approach under direct vision [34]. Moreover, the medial incision ensures a sufficient flap width, which is more conducive to good medial soft tissue coverage. This may reduce the difficulty of wound closure and the possibility of skin necrosis.

Our research believes that staging surgery can ensure the healing of soft tissue. The reduction and fixation of the fibula and the posterior column of the distant tibia can effectively resist valgus stress. In contrast, the distal tibia medial locking plate supports to resist varus pressure. The anterior column was fixed by cannulated screws, effectively avoiding irritation of the nerve, tendons, and blood vessels from locking plates. Hollow screws and distal parallel screws of the medial tibia plate formed a cross-vertical fixing system. The stable fixation of the distal tibial fragments favors patients’ good function through early exercises.

In recent studies, several researchers have suggested that internal fixation of fibular fractures is considered to lack statistically significant evidence in pilon fracture treatment. Despite the controversy, it helped augment external fixation and provide additional stability [40]. In 2021, a study of 76 patients by Hong C. C. et al. noted that patients without fibula fixation have more wound complications (44% vs. 25.9%, *p* = 0.108). A larger sample size may lead to statistically significant conclusions [41]. We believe that, with a large sample size cohort study, the necessity of internal fixation of fibular fractures will be reconsidered. One of the focuses of this surgical technique is to reduce wound complications. We believe that subsequent studies will prove our contention and provide guiding recommendations in the treatment of pilon fractures.

## 5. Conclusions

This is a technique note of the staged treatment of AO/OTA C3 type pilon fracture with fibula fractures. Early reduction and fixation of the lateral and posterior columns could provide a well-repositioned articular surface and alignment. The second-stage posteromedial incision gives a good operative view, while reducing the medial and anterior lateral columns. It ensures early postoperative functional exercise and fewer risks of complications.

## Data Availability

The final dataset will be available from the corresponding author.
